# A Field-Driven Growth Model for Uniform Thin-Film Growth

**DOI:** 10.3390/mi17020220

**Published:** 2026-02-06

**Authors:** Helena Cristina Vasconcelos, Telmo Eleutério, Maria Meirelles

**Affiliations:** 1 Faculty of Science and Technology, University of the Azores, Ponta Delgada, S. Miguel, 9500-321 Azores, Portugal; telmo.mf.eleuterio@uac.pt (T.E.); maria.gf.meirelles@uac.pt (M.M.); 2 Laboratory of Instrumentation, Biomedical Engineering and Radiation Physics (LIBPhys, UNL), Department of Physics, NOVA School of Science and Technology, 2829-516 Caparica, Portugal; 3 Research Institute of Marine Sciences–OKEANOS, University of the Azores, Horta, Faial, 9901-862 Azores, Portugal

**Keywords:** thin-film growth, field-assisted deposition, electric-field effects, surface morphology, field-assisted nucleation, anisotropic surface diffusion, continuum stability, free-energy modelling, sputtering, atomic layer deposition

## Abstract

Externally applied electric fields are widely employed during thin-film deposition to improve film uniformity, texture and densification. Despite extensive experimental evidence, the physical mechanisms by which such fields influence nucleation, surface diffusion, island coalescence and interface stability remain theoretically fragmented. Classical thin-film growth models assume a field-free energetic landscape and therefore provide limited predictive guidance for field-assisted manufacturing strategies. In this work, we introduce the Field-Driven Growth Model (FDGM), a unified theoretical framework that incorporates field–matter interactions directly into the free-energy functional governing thin-film growth. By explicitly accounting for effective dipolar coupling arising from field-induced polarization of surface species, predominantly quadratic in the field amplitude and consistent with linear-response polarization, the model consistently modifies the fundamental processes of nucleation, surface diffusion and coalescence. At the continuum scale, the FDGM predicts a field-induced stabilization mechanism that suppresses long-wavelength roughening modes and defines a field-controlled morphological crossover wavelength (field-controlled cutoff). The FDGM demonstrates that field-assisted nucleation bias, anisotropic surface diffusion, field-biased coalescence pathways and morphological stabilization are not independent phenomena, but multiscale manifestations of a single energy-minimization principle acting on a field-modified energy landscape. By providing analytical stability criteria and explicit links between external field parameters and morphological outcomes, the model establishes a predictive foundation for the manufacturing of thin films with improved uniformity in advanced thin-film-based devices. The framework is broadly applicable to deposition techniques such as sputtering, pulsed-laser deposition, chemical vapor deposition and atomic layer deposition.

## 1. Introduction

The control of thin-film morphology is a central requirement in the manufacturing of advanced thin-film-based devices, where surface roughness, grain orientation and interfacial uniformity directly impact device yield, reproducibility and long-term reliability. In micro- and nanoscale systems—including MEMS, sensors, optoelectronic components and energy devices—the fabrication window is often constrained not by material composition but by the ability to reproducibly control film growth at the nanometer scale [1,2].

Classical thin-film growth theories provide a robust framework for describing film evolution under intrinsic growth conditions. Burton–Cabrera–Frank (BCF) step-flow theory successfully explains the motion of atomic steps and terrace evolution on crystalline surfaces during epitaxial growth [3]. Classical nucleation theory quantitatively relates supersaturation and surface energy to nucleation density and island formation [4]. At larger length scales, Mullins-type surface diffusion models capture curvature-driven interface relaxation and provide the basis for understanding surface smoothening through mass transport [5,6]. These approaches have been widely validated and remain central to thin-film science.

However, a defining characteristic of all these classical frameworks is that the energetic landscape governing growth is assumed to be field-free. External perturbations—when considered at all—are typically introduced indirectly, for example, through effective changes in temperature, mobility or deposition rate. As a result, these models do not provide explicit mechanisms by which external electric fields couple to surface energetics or kinetics.

This limitation becomes evident in modern manufacturing environments. In sputtering and PLD, substrate bias and plasma-induced electric fields are routinely present; in CVD and ALD, electrostatic charging and externally applied fields have been reported to influence nucleation density and film uniformity [1,2]. Numerous experimental studies show that applying external fields can reduce surface roughness, enhance densification and promote preferred crystallographic orientation without modifying chemical precursors or deposition temperature [7,8,9]. Yet these effects cannot be rationalized within BCF, nucleation theory or Mullins-type models, because none of these frameworks contains a term that couples the field directly to surface species.

Existing attempts to address field effects typically rely on phenomenological interpretations. For instance, enhanced smoothening is often attributed to an “effective increase in surface mobility”, while changes in nucleation density are explained post hoc through modified supersaturation or plasma chemistry. While such interpretations may be qualitatively useful, they lack predictive power and fail to explain why similar field-induced effects are observed across fundamentally different deposition techniques.

This gap is particularly evident in continuum growth models. The Edwards–Wilkinson and Kardar–Parisi–Zhang equations describe interface roughening and scaling behavior under stochastic growth conditions [6,10], while the Wolf–Villain model incorporates slope-dependent diffusion to account for mound formation [11]. However, in all these models, anisotropy and instability arise from intrinsic surface properties or nonlinearities, not from externally controllable fields. Consequently, they cannot predict a field-controlled suppression of long-wavelength roughening modes or define stability conditions linked to measurable field parameters.

In this work, we address this unresolved problem by introducing the Field-Driven Growth Model (FDGM). The key novelty of the FDGM is the explicit incorporation of field–matter interactions into the free-energy functional governing thin-film growth. Rather than treating external fields as secondary process parameters, the FDGM shows that effective dipolar coupling between surface species and externally applied electric fields directly reshapes the energetic landscape underlying nucleation, diffusion and coalescence.

As a result, the FDGM provides several capabilities absent from existing approaches. First, it introduces a physically grounded mechanism for field-assisted nucleation through a direct reduction in the nucleation barrier, rather than through indirect changes in supersaturation. Second, it predicts anisotropic surface diffusion arising from directional lowering of diffusion barriers, linking microscopic field effects to mesoscopic growth anisotropy. Third, at the continuum scale, the FDGM yields an explicit field-induced stabilization term in the surface evolution equation, leading to a field-controlled cutoff wavelength beyond which roughening modes are suppressed. None of these features can be derived within classical growth models or phenomenological extensions thereof.

**Figure 1:**
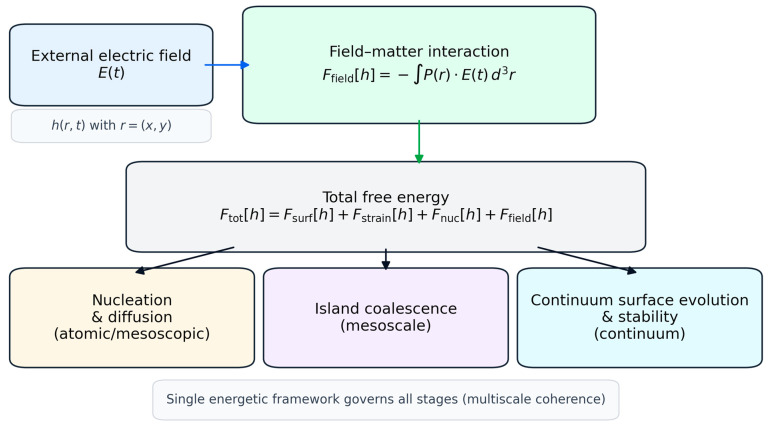
Overview of the Field-Driven Growth Model (FDGM): Schematic representation of the total free-energy functional, $F_{tot}$, highlighting the explicit incorporation of the electric external field through the field–matter interaction term, $F_{field}$. The same energetic framework governs nucleation and diffusion, island coalescence and continuum surface evolution, ensuring multiscale coherence.

By unifying these mechanisms within a single energetic framework, the FDGM fills a critical gap between empirical observations of field-assisted growth and predictive thin-film manufacturing theory. The model explains why field-induced ordering is observed across disparate deposition techniques and provides analytical criteria that directly link external field parameters to morphological outcomes. In doing so, the FDGM establishes a foundation for rational design of field-assisted deposition strategies to target improved surface uniformity in advanced thin-film-based devices. The central concepts and multiscale structure of the FDGM are summarized schematically in Figure 1.

In contrast to existing thin-film growth models, which either neglect external fields or introduce them phenomenologically through modified kinetic coefficients, the FDGM incorporates field–matter interactions directly at the level of the free-energy functional. This variational formulation ensures multiscale consistency across nucleation, diffusion, coalescence and morphological stability, which cannot be achieved within classical or purely kinetic frameworks.

Because the FDGM is derived from first-principles energetic considerations rather than empirical fitting, it does not require experimental calibration to establish qualitative trends and stability regimes, but instead provides analytically testable predictions that can subsequently guide targeted experimental validation. Unlike classical thin-film growth models—including Burton–Cabrera–Frank theory, classical nucleation theory, Mullins-type surface diffusion models, and stochastic continuum approaches such as Edwards–Wilkinson or KPZ—which assume a field-free energetic landscape, the Field-Driven Growth Model explicitly incorporates field–matter interactions into the governing free-energy functional. This formulation allows nucleation, diffusion, coalescence, and continuum-scale morphological stability to be treated within a single variational framework. As a result, the FDGM provides predictive insight into field-assisted growth phenomena without relying on direct experimental fitting or system-specific parameter tuning.

## 2. Field-Driven Energetic Framework

Thin-film growth is governed by the evolution of the system toward configurations that minimize an appropriate free-energy functional under non-equilibrium conditions. In classical growth theories, this functional typically includes contributions from surface and interface energies, elastic strain and, implicitly, chemical driving forces associated with deposition flux and supersaturation [3,4,5]. In this section, we generalize this framework by explicitly incorporating the interaction between externally applied electric fields and surface species into the total free energy.

We consider a growing thin film described by a height field h(r,t), where r=(x,y) denotes the in-plane coordinates. The total free energy is written as

(1) $$F_{tot}[h]=F_{surf}[h]+F_{strain}[h]+F_{nuc}[h]+F_{field}[h].$$

Here, $F_{surf}$ accounts for surface and interface energies, $F_{strain}$ captures elastic contributions arising from lattice mismatch or intrinsic stress, and $F_{nuc}$ represents the energetic cost associated with the formation of stable nuclei and islands, as described within classical nucleation theory [4]. These terms constitute the standard energetic description underlying BCF step-flow models, continuum surface diffusion theories and related approaches [3,5,6].

The defining extension introduced in the Field-Driven Growth Model (FDGM) is the explicit inclusion of a field–matter interaction term,

(2) $$F_{field}[h]=-\frac{1}{2}\int P(r)\cdot E(t)\;d^{3}r,$$

where E(t) denotes an externally applied electric field and P(r) is the local polarization density associated with surface adatoms, small clusters, or islands. Such dipolar coupling terms are well established in condensed-matter descriptions of field–matter interactions and provide a physically transparent route to incorporate external fields into the energetic landscape [12,13]; they have also been widely invoked in the context of field-assisted thin-film growth [1,2].

For polarizable surface species, the polarization can be expressed to leading order as

(3) $$P(r)=\alpha_{E}\;E(t),$$

where $\alpha_{E}$ is an effective polarizability that depends on the chemical nature of the adsorbate and its local environment, as described in classical linear-response theory [12,13]. In the linear-response regime, Equation (2) recovers the familiar induced-dipole energy scaling $\Delta U∼-\frac{1}{2}\alpha E^{2}$. In this formulation, the polarization is assumed to be predominantly induced rather than permanent, which is appropriate for the moderate field strengths relevant to thin-film manufacturing, where higher-order nonlinear polarization effects can be neglected.

Accordingly, the associated field–matter interaction energy for induced polarization is fundamentally quadratic in the electric field amplitude, $\Delta U∼-\frac{1}{2}\alpha_{E}E^{2}$. Any effective linear dependence on the field appearing in subsequent sections should, therefore, be understood as arising from energy differences between metastable and transition (saddle-point) configurations along activated pathways, reflecting geometric asymmetry and induced charge redistribution, rather than from a fundamental linear dipole–field coupling

The temporal evolution of the film surface is assumed to follow gradient dynamics driven by the functional derivative of the total free energy,

(4) $$\frac{\partial h(r,t)}{\partial t}=-M\frac{\delta F_{tot}}{\delta h(r,t)}+\eta (r,t),$$

where M is an effective mobility and η(r,t) represents stochastic fluctuations arising from deposition noise and thermal effects. This form is consistent with continuum descriptions of surface evolution under surface-diffusion-limited kinetics [5,6], and provides the baseline relaxation dynamics upon which growth-specific terms are later discussed.

The presence of $F_{field}$ in Equation (4) constitutes the central conceptual advance of the FDGM. Unlike classical growth models, where external perturbations enter only indirectly through modified kinetic coefficients, the FDGM introduces the external field directly into the energetic driving force. As a consequence, nucleation barriers, diffusion pathways, island interactions and large-scale morphological stability are all modified in a consistent and physically grounded manner.

It is important to emphasize that Equation (2) does not assume a specific deposition technique or field configuration. The framework applies equally to static or time-dependent electric fields and is, therefore, relevant to a wide range of manufacturing environments, including substrate-biased sputtering, plasma-assisted PLD and field-influenced CVD or ALD processes [1,2,8]. The subsequent sections demonstrate how this single additional energetic term propagates across length scales, yielding field-assisted nucleation (Section 3), anisotropic surface diffusion (Section 4), directed island coalescence (Section 5) and field-induced stabilization of surface modes (Section 6).

### Orders of Magnitude and Regime of Applicability

Although the Field-Driven Growth Model (FDGM) is formulated in a general manner, it is important to establish representative orders of magnitude for the relevant parameters in order to clarify the physical regime in which the predicted effects become relevant for realistic thin-film deposition processes.

For electric fields applied during field-assisted growth, typical values range from $E∼10^{4}$–$10^{6}\;V\;m^{-1}$, as commonly encountered in substrate biasing, plasma-enhanced atomic layer deposition, and field-assisted chemical vapor deposition environments [8,14,15,16]. The effective polarizability of surface adatoms or small clusters is typically of the order $\alpha ∼10^{-40}$–$10^{-38}\;C\;m^{2}\;V^{-1}$, depending on the chemical nature of the species and its local bonding environment [12,13]. Within these ranges, the induced-dipole contribution $\Delta U∼-\frac{1}{2}\alpha E^{2}$ is generally small compared with bond energies, but it can be comparable to the free-energy differences between competing activated pathways and therefore measurably bias nucleation statistics and early-stage morphology.

While this energy scale is small compared to typical atomic bond energies, it is comparable to $k_{B}T$ at moderate growth temperatures (300–600 K) and, more importantly, to the energetic differences between competing activated pathways in nucleation and surface diffusion. Recent experimental and modelling studies have shown that electric fields of this magnitude can measurably alter surface reaction energetics, nucleation density and film morphology without modifying chemical precursors or growth temperature [14,15]. Consequently, even modest field-induced energetic contributions can have a statistically significant impact on rare-event selection and collective surface evolution.

The FDGM explicitly assumes this regime of moderate field strengths, in which linear polarization provides an adequate description of field–matter interactions [12,13] and nonlinear effects such as field-induced desorption, surface ionization or strong electronic restructuring can be neglected. Extension of the model to extreme-field regimes would require additional energetic terms beyond the scope of the present framework.

To guide the reader before we move on to the multiscale formulation, Table 1 summarizes the key FDGM parameters used throughout the paper, their physical meaning, units/scaling in the model, and practical routes for experimental inference.

## 3. Field-Assisted Nucleation

Nucleation constitutes the first irreversible step in thin-film growth and plays a decisive role in determining island density, spatial uniformity and the subsequent evolution of film morphology. Within classical nucleation theory, the formation of stable clusters results from a competition between the energetic cost of creating new interfaces and the thermodynamic driving force associated with supersaturation or chemical potential difference [4].

### 3.1. Classical Nucleation Barrier

For a cluster of characteristic radius r, the classical free-energy change can be written as

(5) $$G(r)=4\pi r^{2}\gamma -\frac{4}{3}\pi r^{3}\Delta \mu ,$$

where γ is an effective surface energy, and Δμ denotes the chemical potential difference driving growth. The critical radius $r_{c}$ and the corresponding nucleation barrier $\Delta G^{*}$ follow from the extremum condition ∂G/∂r=0, yielding

(6) $$r_{c}=\frac{2\gamma }{\Delta \mu },\Delta G^{*}=\frac{16\pi \gamma^{3}}{3(\Delta \mu {)}^{2}}.$$

The nucleation rate is then given by

(7) $$J=J_{0}exp\left(-\frac{\Delta G^{*}}{k_{B}T}\right),$$

where $J_{0}$ is a kinetic pre-factor. This framework successfully describes nucleation under field-free conditions and has been extensively validated across a wide range of thin-film systems [4,6].

### 3.2. Modification of the Nucleation Barrier by External Fields

In the presence of an external electric field, surface adatoms and small clusters may acquire an effective dipole moment due to intrinsic polarity or, more commonly, induced polarization. Within the FDGM introduced in Section 2, this interaction contributes an additional energetic term associated with the coupling between the cluster polarization and the external field.

At the level of the free-energy functional, the fundamental field-dependent contribution for induced dipoles is quadratic in the electric field, of the form $\Delta U=-\frac{1}{2}\alpha E^{2}$.

However, when evaluating the stability of a given cluster configuration relative to the activated nucleation pathway, the difference in field-modified energy between the saddle-point configuration and the metastable state introduces an effective directional bias. This bias reflects the asymmetry of the nucleation pathway and saddle-point geometry under the applied field, rather than a true linear dipole–field coupling.

Accordingly, the field-related contribution to the cluster free energy difference relevant for nucleation can be written, to leading order, as

(8) $$G_{field}=-\;p_{eff}\cdot E,$$

where $p_{eff}$ denotes an effective dipolar projection parameter that encapsulates the quadratic polarization response, the geometry of the critical nucleus and induced charge redistribution along the applied field direction, and E is the external electric field.

The total free energy associated with cluster formation thus becomes

(9) $$G_{tot}(r)=G(r)-p_{eff}\cdot E.$$

To leading order, the presence of the field does not significantly alter the critical radius, $r_{c}$, in regimes relevant to thin-film manufacturing, but it does modify the height of the nucleation barrier. Evaluated at $r=r_{c}$, the effective barrier reads

(10) $$\Delta G_{eff}^{*}=\Delta G^{*}-\lambda (p_{eff}\cdot E),$$

where λ is a geometric factor that accounts for cluster shape, partial wetting and orientation effects [1,4].

This formulation should be understood as an effective barrier-bias representation of field-induced asymmetry between competing nucleation pathways, rather than as a fundamental linear field–matter interaction for an induced dipole in a strictly uniform field. In practice, the activation barrier depends on differences in polarization response and/or effective dipole moment between the metastable configuration and the transition-state configuration; therefore, the field-induced contribution can be recast, to leading order, into a directional term proportional to $p_{eff}\cdot E$ (see Appendix A).

### 3.3. Field-Assisted Nucleation Rate and Physical Implications

Substituting Equation (10) into Equation (7), the nucleation rate in the presence of a field becomes

(11) $$J=J_{0}exp\left[-\frac{\Delta G^{*}-\lambda (p_{eff}\cdot E)}{k_{B}T}\right].$$

This expression highlights several important physical consequences absent from classical nucleation theory:

- Barrier reduction without chemical modification.
  The nucleation barrier is lowered directly by the field–matter interaction, without requiring changes in supersaturation, precursor chemistry or substrate temperature. This provides a natural explanation for experimentally observed increases in nucleation density under applied electric fields in sputtering, PLD and ALD processes [1,2].
- Directional bias in nucleation.
  Because the barrier reduction depends on the projection $p_{eff}\cdot E$, nucleation becomes orientation-dependent. Clusters aligned with the field are energetically favored, leading to spatially more uniform and directionally correlated nucleation patterns.
- Enhanced reproducibility.
  By reducing the sensitivity of J to stochastic fluctuations in Δμ, the field-assisted term narrows the distribution of nucleation events. This effect is particularly relevant for manufacturing contexts where reproducibility and uniformity are critical.

It is important to emphasize that the effective dipole moment $p_{eff}$ introduced here does not require the presence of permanently polar species. In many technologically relevant systems, polarization arises from an induced response of adatoms or small clusters to the external field and to the electronic environment of the substrate, as described within classical electrodynamics of condensed media [12,13].

This mechanism is particularly relevant for semiconductors, oxides and partially ionic surfaces, where field-induced charge redistribution can generate transient dipole moments sufficient to influence energetic barriers. Recent studies on oxide and dielectric thin films have directly demonstrated electric-field-induced modifications of nucleation behavior [9,14] and field-assisted phase stability effects in thin-film systems [17], consistent with the energetic mechanisms discussed here. In metallic systems, although individual adatom polarization is strongly screened, collective effects associated with clusters, interfaces or near-surface field gradients may still yield non-negligible contributions at the statistical level relevant for nucleation and early-stage growth [1,7].

Accordingly, the FDGM does not rely on a restrictive material-specific assumption but rather captures a generic mechanism applicable whenever an effective polarizable response of surface species is present.

### 3.4. Relation to Existing Approaches and Unresolved Limitations

Previous attempts to rationalize field-assisted nucleation often invoke indirect mechanisms, such as effective changes in surface mobility, local temperature or plasma chemistry. While such interpretations may capture specific experimental trends, they do not provide an explicit energetic mechanism linking the external field to the nucleation barrier itself.

In contrast, the FDGM introduces this link directly at the level of the free-energy functional. The effective linear bias appearing in Equation (10) should be understood as a leading-order manifestation of an underlying field-dependent energy landscape, ensuring consistency with the induced-polarization framework introduced in Section 2. Importantly, this mechanism cannot be derived within classical nucleation theory or through phenomenological extensions that treat the field solely as a kinetic modifier.

The consequences of the reduced and direction-dependent nucleation barrier propagate to later growth stages. Increased nucleation density and spatial regularity influence adatom capture, island growth and eventual coalescence, providing a natural bridge to the field-induced diffusion anisotropy discussed in Section 4.

## 4. Field-Induced Surface Diffusion

Following nucleation, surface diffusion governs mass transport across the growing interface and plays a central role in determining island growth kinetics, coalescence pathways and the development of surface roughness. In classical growth theory, surface diffusion is treated as a thermally activated process driven by gradients in chemical potential, with isotropic diffusion barriers that depend solely on the local atomic environment [5,6].

### 4.1. Classical Description of Surface Diffusion

Under field-free conditions, the surface diffusion coefficient is commonly expressed as

(12) $$D=D_{0}exp\left(-\frac{E_{d}}{k_{B}T}\right),$$

where $D_{0}$ is a prefactor related to the attempt frequency and jump distance, $E_{d}$ is the activation energy for adatom hopping, $k_{B}$ is the Boltzmann constant and T is the substrate temperature. This formulation underlies both atomistic models and continuum descriptions of surface-diffusion-limited growth, including Mullins-type relaxation theories [5,6].

Within this framework, diffusion is intrinsically isotropic on sufficiently symmetric surfaces, and any anisotropy arises from crystallographic effects, step-edge barriers or surface reconstruction. External fields do not explicitly appear in the energetic description and therefore cannot directly bias diffusion pathways.

### 4.2. Modification of the Diffusion Barrier by External Fields

In the FDGM, surface diffusion is affected by the same field–matter interaction introduced in Section 2. Adatoms migrating across the surface experience a local energetic contribution arising from their interaction with an external electric field through induced polarization effects.

At the level of the free-energy functional, the field-dependent contribution to the adatom energy is quadratic in the electric field. However, during a thermally activated diffusion event, the relevant quantity is the energy difference between the initial adsorption site and the transition (saddle-point) configuration. The asymmetry of these configurations with respect to the field direction gives rise to an effective directional bias. This bias reflects differences in induced polarization and charge redistribution between metastable and saddle-point configurations, rather than a fundamental linear dipole–field interaction.

For a diffusion hop characterized by a direction θ relative to the applied field, the effective activation energy can, therefore, be written as

(13) $$E_{d,eff}(\theta )=E_{d}-\beta |E|cos\theta ,$$

where β is a coupling coefficient that depends on the polarizability of the diffusing species and the local surface environment. This expression represents a linearized form of the field-induced energetic asymmetry between initial and transition states, valid in the regime of moderate fields and small activation-energy differences, and should not be interpreted as implying a true linear dependence of the total polarization energy on the field amplitude. Diffusion hops aligned with the field direction experience a reduced effective barrier, while hops opposing the field are energetically disfavored.

The resulting diffusion coefficient becomes explicitly anisotropic,

(14) $$D(\theta )=D_{0}exp\left[-\frac{E_{d}-\beta |E|cos\theta }{k_{B}T}\right].$$

Such anisotropic diffusion and enhanced directional mass transport have been reported experimentally in energetically assisted and field-assisted growth environments, where preferential mass transport along selected directions leads to smoother surfaces and more uniform island growth [1,2,18].

### 4.3. Physical Implications of Anisotropic Diffusion

The directional dependence introduced in Equation (14) has several important consequences:

- Enhanced mass transport along field-favored directions.
  Adatoms preferentially migrate along directions that minimize the effective diffusion barrier, leading to accelerated filling of low-lying regions and suppression of height fluctuations.
- Coupling between nucleation density and diffusion length.
  The increased nucleation density predicted in Section 3 reduces the average diffusion length, while anisotropic diffusion redistributes mass more efficiently between neighbouring islands. Together, these effects promote homogeneous growth and reduce the likelihood of isolated, fast-growing features.
- Suppression of kinetic roughening.
  By biasing diffusion toward configurations that reduce the total free energy, within an otherwise conserved surface-diffusion framework, field-induced anisotropy counteracts the stochastic amplification of surface roughness typically observed under non-equilibrium deposition conditions.

### 4.4. Relation to Existing Diffusion Models

Slope-dependent diffusion models, such as the Wolf–Villain model, incorporate anisotropy through intrinsic surface geometry and local slope effects [11]. While these approaches successfully describe mound formation and kinetic roughening in certain regimes, the anisotropy they predict is not externally tunable and arises solely from surface morphology.

In contrast, the FDGM introduces externally controllable anisotropy through the applied field. The diffusion bias in Equation (14) does not depend on surface slope or crystallography alone, but can be modulated dynamically by adjusting field strength or orientation. This distinction is crucial for manufacturing applications, where external control parameters are preferred over intrinsic material constraints.

Moreover, the anisotropic diffusion derived here emerges from the same field-modified free-energy functional that governs nucleation and, as shown in the following section, island coalescence. This ensures energetic consistency across growth stages, which is absent in phenomenological extensions of classical diffusion models.

### 4.5. Transition to Mesoscale Coalescence

As islands grow and their diffusion fields overlap, the anisotropic transport of adatoms leads naturally to biased coalescence pathways. Regions that minimize the combined surface, strain and field energy are preferentially filled, driving islands toward energetically favorable configurations. In this sense, directed coalescence emerges as a collective consequence of biased mass transport rather than as a separate force-driven mechanism, providing the physical basis for the processes discussed in Section 5.

## 5. Directed Coalescence of Islands

As thin-film growth progresses beyond the initial nucleation and diffusion stages, individual islands expand, interact and eventually coalesce to form a continuous film. The manner in which islands coalesce strongly influences grain size distribution, texture development and defect formation. In classical growth models, coalescence is largely governed by geometric proximity and isotropic mass transport, with limited control over the pathways by which islands merge [2,18].

### 5.1. Classical View of Island Coalescence

Under field-free conditions, island coalescence is typically described as a consequence of surface diffusion and capture-zone overlap. As islands grow, their diffusion fields intersect, leading to material transfer that fills the gaps between neighbouring features. This process is often treated as isotropic and driven by local curvature and chemical potential gradients, without invoking long-range interactions or directional forces [5,6].

While such descriptions successfully account for basic coalescence kinetics, they provide little insight into experimentally observed texture selection, grain alignment or enhanced densification under field-assisted growth conditions. In particular, classical models cannot explain why islands appear to merge preferentially along specific directions or why certain grain orientations are stabilized during coalescence.

### 5.2. Field-Induced Energetic Bias on Islands

Within the FDGM, islands are treated as mesoscale entities characterized by an effective polarization $P_{isl}$, arising from the collective response of their constituent atoms to the external field. The interaction energy between an island and the field can be written as

(15) $$F_{field}(R)=-P_{isl}(R)\cdot E,$$

where R denotes the position of the island’s centre of mass.

In a strictly uniform external field, this term does not generate a net translational force on an isolated island. However, it modifies the energetic cost of island configurations, shapes and orientations, and therefore biases the energetically favorable pathways through which islands exchange mass and evolve during coalescence.

Spatial variations in island polarization, local surface morphology or near-surface field gradients—such as those naturally present close to island edges, steps or plasma sheaths—can further enhance this energetic bias without requiring a macroscopic field gradient.

Accordingly, the role of the field at the mesoscale is not to exert a classical mechanical force on islands, but to reshape the effective energy landscape that governs their interaction and mass-transfer pathways.

This energetic bias derives from the same field–matter coupling introduced at the atomistic scale, ensuring consistency between microscopic diffusion anisotropy and mesoscale island dynamics.

### 5.3. Directed Coalescence and Texture Selection

The presence of a field-induced energetic bias modifies the landscape governing island interactions. Coalescence no longer occurs solely along the shortest geometric path, but preferentially follows trajectories that minimize the combined surface, strain and field energy. As a result:

- Directional merging of islands.
  Islands tend to coalesce along field-favored directions, leading to anisotropic grain shapes and aligned grain boundaries.
- Enhanced densification.
  Field-biased mass transport promotes the elimination of voids and narrow gaps that would otherwise persist during isotropic coalescence.
- Texture stabilization.
  Grain orientations that couple favorably to the external field are selectively stabilized during coalescence, providing a mechanism for field-induced texture control observed in sputtering and PLD processes [8,18].

These effects provide a natural explanation for experimental observations of improved film density and orientation under applied fields, even in the absence of a net force acting on individual islands, and cannot be readily explained by diffusion anisotropy alone.

### 5.4. Relation to Existing Mesoscale Models

Mesoscale coalescence models typically treat islands as passive geometric entities whose interactions are mediated solely by surface diffusion and elastic strain [2,18]. While such approaches can capture coarsening dynamics, they do not include external control parameters capable of biasing coalescence pathways.

In contrast, the FDGM introduces an externally controllable energetic contribution that directly influences island interactions. This distinction is particularly important for manufacturing applications, where control over texture and defect density is often more critical than equilibrium grain size.

### 5.5. Transition to Continuum-Scale Morphology

Directed coalescence represents the final mesoscale manifestation of the field-modified energy landscape. Once islands have merged into a continuous film, the cumulative effects of field-assisted nucleation, anisotropic diffusion and energetically biased coalescence manifest as large-scale morphological changes. In the following section, we formalize this connection by deriving a continuum surface evolution equation that captures the field-induced stabilization of interface modes.

## 6. Continuum Surface Evolution and Stability

Once island coalescence has produced a laterally continuous film, thin-film growth can be described at the continuum level by the evolution of the surface height field h(r,t). At this stage, the cumulative effects of field-assisted nucleation, anisotropic diffusion and directed coalescence manifest as modifications to the stability and relaxation behavior of surface modes. In this section, we derive the continuum evolution equation within the FDGM framework and analyze its implications for morphological stability.

### 6.1. Classical Continuum Description

Under surface-diffusion-limited kinetics, the temporal evolution of the surface height is governed by mass conservation,

(16) $$\frac{\partial h}{\partial t}=-\nabla \cdot J_{s},$$

where $J_{s}$ is the surface mass flux. In the continuum description, curvature-driven surface transport is represented by a surface flux driven by gradients of the chemical potential,

(17) $$J_{s}=-M_{s}\nabla \mu ,$$

where $M_{s}$ is an effective surface mobility. For small surface slopes, the chemical potential is proportional to the local curvature,

(18) $$\mu =\gamma \nabla^{2}h,$$

where γ is the surface energy. Substituting Equations (17) and (18) into Equation (16) yields the classical Mullins equation,

(19) $$\frac{\partial h}{\partial t}=-\nu \nabla^{4}h,$$

where ν (units $m^{4}\;s^{-1}$) is the Mullins surface-smoothing coefficient. In many surface-diffusion models, $M_{s}$ (and hence ν) can be related to microscopic quantities such as the surface diffusivity $D_{s}$, the atomic volume Ω, and the near-surface atomic density; however, for the purposes of the present continuum treatment, we retain $M_{s}$ and ν as effective coefficients. This equation describes curvature-driven relaxation of surface roughness through surface diffusion [5,6].

Fourier decomposition of the surface profile,

(20) $$h(r,t)=\sum_{k}h_{k}(t)\;e^{ik\cdot r},$$

leads to the classical dispersion relation

(21) $$\omega_{k}^{\left(0\right)}=-\nu k^{4},$$

indicating that all surface modes decay in time, albeit with very slow relaxation for long-wavelength modes (k→0).

It is important to note that while Equations (16)–(21) describe purely conservative surface-diffusion-limited relaxation, real thin-film growth typically involves additional non-conservative processes, such as attachment–detachment kinetics, surface incorporation and exchange with the deposition flux. In such regimes, the surface height is not strictly conserved locally, and effective non-conservative relaxation terms may arise in the continuum description, consistent with Edwards–Wilkinson-type dynamics.

### 6.2. Field-Induced Modification of the Continuum Dynamics

To account for the coexistence of surface-diffusion-driven mass transport and attachment–detachment kinetics during thin-film growth, we adopt a generalized continuum description combining conservative and non-conservative relaxation channels. The surface height evolution is, therefore, written in the canonical form

(22) $$\frac{\partial h}{\partial t}=\nabla \cdot \left(M\nabla \mu \right)-L\;\mu +\eta (r,t)$$

where $\mu =\delta F_{tot}/\delta h$, the first term represents conservative surface diffusion, while the second term accounts for non-conservative relaxation associated with attachment–detachment and incorporation processes during growth.

Within the FDGM, the surface chemical potential derives from the functional derivative of the total free energy defined in Section 2. At the continuum stage of growth, once nucleation has ceased and the film is laterally continuous, only those free-energy contributions that depend explicitly on the surface height field contribute to the chemical potential. In this regime, the nucleation term no longer depends on h, while elastic strain contributions are assumed to be either constant or weakly dependent on h and are therefore absorbed into effective material parameters. As a result, the chemical potential reduces to

(23) $$\mu =\frac{\delta F_{tot}}{\delta h}=\frac{\delta F_{surf}}{\delta h}+\frac{\delta F_{field}}{\delta h}.$$

For small height fluctuations around a nominally flat surface, local variations in h(x,y) modify the spatial distribution and density of polarizable surface species exposed to the external field, and therefore the integrated dipolar interaction energy. Expanding the field-dependent contribution about the flat reference state and retaining terms linear in the surface height yields, to leading order, a restoring contribution proportional to the local height deviation h.

In the presence of attachment–detachment kinetics or exchange with the deposition flux, this restoring contribution enters the continuum dynamics as an effective non-conservative relaxation term, reflecting the energetic penalty associated with deviations from configurations that minimize the field–matter interaction energy.

Higher-order contributions become relevant only for large amplitudes or steep surface slopes and are neglected here, as the present stability analysis is restricted to the regime of small perturbations, consistent with classical continuum treatments of surface diffusion and morphological relaxation [4,5,11].

The surface energy contribution recovers the classical curvature term in Equation (18). The field-dependent contribution introduces an additional restoring tendency that penalizes deviations of the surface profile from configurations that minimize the field–matter interaction energy. Accordingly, the field-induced contribution acts as an effective non-conservative stabilizing channel in the continuum evolution. This contribution can be expressed as a linear stabilizing term,

(24) $${\frac{\partial h}{\partial t}|}_{field}=-\Gamma_{AC}\;h,$$

where $\Gamma_{AC}$ is a positive coefficient that quantifies the strength of the field-induced stabilization. It is important to distinguish between microscopic kinetic anisotropy and macroscopic morphological stability. While the applied field biases atomic diffusion and attachment pathways along energetically favored directions, the suppression of long-wavelength surface roughness derived in Section 6 arises from an effective non-conservative energetic damping term acting at the continuum scale. As a result, the stabilization of surface modes is isotropic in wavevector space, even though the underlying diffusion kinetics are directionally biased.

Physically, $\Gamma_{AC}$ represents the rate at which field-modified energetics penalize local height deviations through attachment–detachment and incorporation processes, rather than through purely diffusive mass transport. For time-dependent or alternating fields, $\Gamma_{AC}$ scales with the time-averaged field intensity and the effective polarizability of the surface species, consistent with experimental and theoretical studies of field- and energy-assisted growth processes [1,2].

The resulting continuum evolution equation becomes

(25) $$\frac{\partial h}{\partial t}=-\nu \nabla^{4}h-\Gamma_{AC}h+\eta (r,t),$$

where ν (units $m^{4}\;s^{-1}$) is the Mullins surface-smoothing coefficient (prefactor of the curvature-driven $\nabla^{4}h$ term), $\Gamma_{AC}$ is the field-induced relaxation rate, and η(r,t) represents stochastic noise associated with deposition and thermal fluctuations. In microscopic surface-diffusion models, ν can be related to $D_{s}$, γ, Ω, T, and the near-surface atomic density; here it is treated as an effective coefficient [5,6].

### 6.3. Dispersion Relation and Stability Spectrum

Applying the Fourier decomposition in Equation (21) to Equation (25) yields

(26) $$\frac{dh_{k}}{dt}=-\left(\nu k^{4}+\Gamma_{AC}\right)h_{k}+\eta_{k}(t).$$

The corresponding dispersion relation is therefore

(27) $$\omega_{k}=-\nu k^{4}-\Gamma_{AC}$$

which constitutes a central result of the FDGM in the regime where non-conservative surface kinetics coexist with surface diffusion during growth. Compared to the classical Mullins spectrum in Equation (21), the field-induced term introduces a uniform downward shift in all surface modes. Because the dispersion relation depends only on the magnitude of the wavevector, the continuum stability spectrum predicted by Equation (27) is isotropic by construction and does not encode directional roughness effects.

For clarity, one may consider a generalized linear representation in which anisotropic surface diffusion enters through an angular-dependent conservative contribution, while the field-induced non-conservative relaxation remains isotropic. In such a schematic form, the growth rate may be written as $\omega_{k}-\nu (\phi )k^{4}-\Gamma_{AC}$, where ϕ denotes the in-plane orientation of the wavevector *k* and ν(ϕ) captures possible diffusion anisotropy arising from biased surface transport. Essentially, $\Gamma_{AC}$ is independent of ϕ and therefore produces a uniform downward shift in the dispersion spectrum for all orientations. The stabilization mechanism discussed here is thus isotropic in wavevector space, even though the underlying microscopic diffusion and attachment kinetics may remain directionally biased by the applied field.

**Figure 2:**
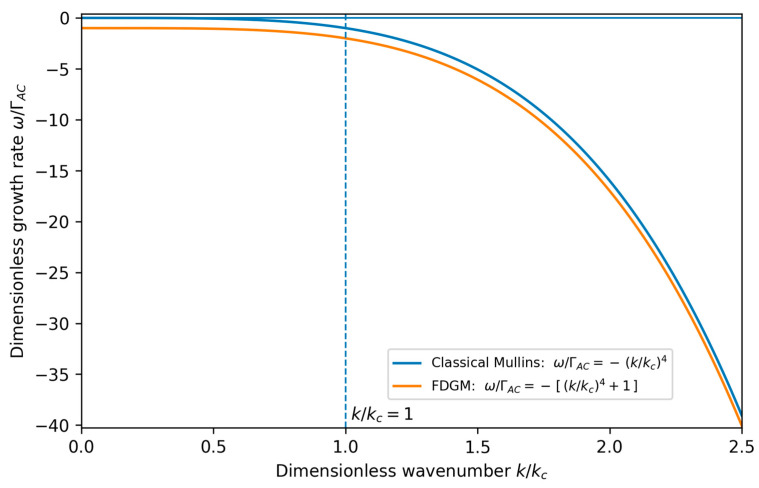
Continuum stability spectrum in the Field-Driven Growth Model: Comparison between the classical Mullins dispersion relation, $\omega_{k}=-\nu k^{4}$, and the field-modified spectrum predicted by the FDGM, $\omega_{k}=-\nu k^{4}-\Gamma_{AC}$. The field-induced term produces a uniform downward shift in the spectrum, leading to enhanced damping of long-wavelength modes (small k) while preserving the curvature-dominated behavior at large k.

The resulting dispersion relation is illustrated schematically in Figure 2, which highlights the uniform downward shift in the stability spectrum induced by the field-dependent term and the enhanced damping of long-wavelength surface modes.

The field-induced contribution does not introduce a new instability but instead leads to a constant downward shift in the dispersion spectrum, characteristic of a stabilizing non-conservative relaxation channel, so that the FDGM growth rate remains more negative than the classical Mullins prediction for all wave numbers.

### 6.4. Field-Controlled Stability Cutoff

The relative importance of curvature-driven relaxation and field-induced stabilization depends on the wavenumber k. Defining a characteristic cutoff wavenumber $k_{c}$ through the condition

(28) $$\nu k_{c}^{4}=\Gamma_{AC},$$

we obtain

(29) $$k_{c}={\left(\frac{\Gamma_{AC}}{\nu }\right)}^{1/4}.$$

This cutoff, therefore, represents a crossover between diffusion-dominated relaxation at short wavelengths and field-controlled non-conservative damping at long wavelengths, rather than a sharp instability threshold.

For modes with $k<k_{c}$, the decay rate is dominated by the field-induced term, and long-wavelength roughening modes are strongly suppressed. In contrast, modes with $k>k_{c}$ relax primarily through classical curvature-driven diffusion. The associated relaxation time is

(30) $$\tau_{k}=\frac{1}{\nu k^{4}+\Gamma_{AC}}.$$

In the regime $\Gamma_{AC}\gg \nu k^{4}$, the decay time becomes approximately independent of wavelength, consistent with the behavior of strongly damped surface modes in classical continuum descriptions of surface relaxation [5,6].

### 6.5. Implications for Morphological Control

The existence of a field-controlled cutoff wavelength represents a qualitative departure from classical growth theory, in which long-wavelength surface modes are only weakly relaxed by curvature-driven diffusion [5,6]. In field-free systems, long-wavelength modes relax extremely slowly and dominate surface roughness at late stages of growth [5,6]. Within the FDGM, these modes are actively damped by the external field, providing a direct mechanism for suppressing large-scale roughness.

**Figure 3:**
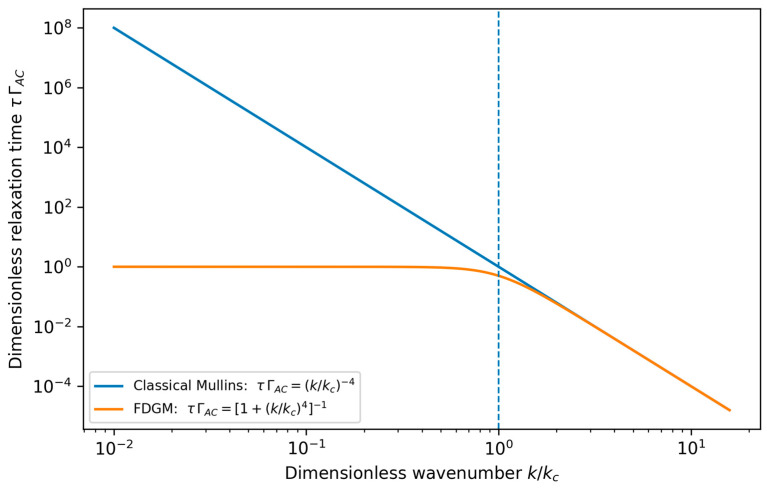
Field-induced acceleration of long-wavelength relaxation in the FDGM: Dimensionless relaxation time $\tau \;\Gamma_{AC}$ as a function of the dimensionless wavenumber $k/k_{c}$, comparing the classical Mullins limit (curvature-driven relaxation, $\tau \;\Gamma_{AC}\propto (k/k_{c}{)}^{-4}$) with the FDGM prediction $\tau \;\Gamma_{AC}=[1+(k/k_{c}{)}^{4}{]}^{-1}$. The dashed line marks the crossover scale $k_{c}=(\Gamma_{AC}/\nu {)}^{1/4}$ separating diffusion-dominated relaxation at short wavelengths ($k\gg k_{c}$) from field-controlled damping at long wavelengths ($k\ll k_{c}$). In the FDGM, long-wavelength modes relax on a timescale of order $\Gamma_{AC}^{-1}$, avoiding the slow large-scale relaxation characteristic of the classical Mullins regime.

To make this effect explicit, Figure 3 compares the mode-dependent relaxation time τ(k) in the classical Mullins limit and in the FDGM. While curvature-driven relaxation yields $\tau \propto k^{-4}$ and therefore extremely slow smoothing at long wavelengths, the FDGM introduces a finite field-controlled timescale such that τ(k) remains bounded by $O(\Gamma_{AC}^{-1})$ for $k\ll k_{c}$. This provides a compact quantitative interpretation of the field-controlled “stability horizon” discussed above.

Importantly, the stability condition expressed by Equations (27)–(29) links morphological control to measurable process parameters through ν and $\Gamma_{AC}$. This connection enables predictive tuning of deposition conditions to achieve uniform surfaces, a capability absent from classical continuum models and phenomenological extensions thereof.

The stabilization mechanism derived here constitutes the macroscopic manifestation of the same field-modified energetic landscape that governs nucleation, diffusion and coalescence at smaller scales. In the following section, we demonstrate how these processes can be unified within a single energy-minimization argument spanning all relevant length scales.

The stabilization mechanism derived here is thus consistent with experimentally relevant growth regimes, where surface diffusion, attachment–detachment and field-modified energetics act simultaneously.

#### Illustrative Example of Field-Induced Stabilization

To illustrate the quantitative impact of the field-induced stabilization term, consider a system with surface energy $\gamma ∼1\;J\;m^{-2}$, an effective surface diffusion coefficient $D_{s}∼10^{-12}\;m^{2}\;s^{-1}$, and an applied field such that the field-induced relaxation rate satisfies $\Gamma_{AC}∼10^{-2}$–$10^{-1}\;s^{-1}$. These values are consistent with orders of magnitude reported in recent field-assisted deposition studies and modelling of electric-field-driven growth processes [8,14,15,16].

Under these conditions, the cutoff wavelength $\Lambda_{c}=2\pi /k_{c}$ lies in the range of several tens to a few hundreds of nanometres, indicating that morphological fluctuations at length scales relevant for micro- and nanoscale devices can be efficiently damped during growth. This simplified example demonstrates how moderate external fields can exert substantial morphological control without altering growth temperature or chemical composition.

The FDGM does not aim to provide quantitative predictions of absolute roughness amplitudes or growth rates for a specific experimental setup. Instead, its reliability lies in predicting robust qualitative trends, stability regimes and crossover length scales. In particular, the existence of a field-controlled cutoff wavelength and the systematic suppression of long-wavelength modes constitute predictions that are insensitive to microscopic details and are therefore expected to be reproducible across different deposition platforms. Accordingly, when applied in a laboratory context, the FDGM is expected to provide high confidence in the direction and relative magnitude of field-induced morphological changes, rather than precise quantitative values, which depend on system-specific kinetic parameters. Although the FDGM does not aim at exact quantitative prediction of surface morphology, it provides robust and internally consistent criteria to assess when and how external fields can suppress roughening, bias transport and stabilize thin-film growth.

## 7. Unified Energy-Minimization Argument Linking Nucleation, Diffusion, Coalescence and Stability

The analyses presented in Section 3, Section 4, Section 5 and Section 6 demonstrate that external fields influence thin-film growth across multiple length scales, from atomic nucleation events to continuum-scale surface stability. In this section, we show that these effects are not independent or phenomenological, but rather arise as consistent manifestations of a single underlying principle: the minimization of a field-modified free-energy functional.

### 7.1. Variational Origin of Field-Driven Growth

Within the FDGM, thin-film evolution is governed by the total free energy

$$F_{tot}=F_{surf}+F_{strain}+F_{nuc}+F_{field},$$

where the defining addition is the explicit field–matter interaction term $F_{field}$. The system evolves under non-equilibrium conditions, but its kinetics are still constrained to follow directions that reduce the instantaneous free energy, subject to deposition flux and thermal noise, and allowing for both conservative and non-conservative kinetic channels, consistent with variational and gradient-flow descriptions of surface evolution in non-equilibrium systems [5,6,12]. The gradient-dynamics form introduced in Section 2 ensures that the functional derivative of $F_{tot}$ acts as the unifying energetic driving force within the present framework.

From this perspective, the role of the external field is not to introduce a new, independent mechanism at each stage of growth, but to reshape the effective stability spectrum in which all growth processes take place. At the continuum scale, this reshaping appears explicitly as an additional non-conservative damping channel in the linear dispersion relation, producing a uniform downward shift in the growth rates and a pronounced enhancement of long-wavelength relaxation (small k). A direct consequence is that the relaxation time of large-scale surface modes becomes bounded by the field-controlled rate scale, rather than diverging as in purely curvature-driven Mullins relaxation. This field-induced acceleration of long-wavelength smoothing provides a compact quantitative interpretation of the “stabilization horizon” introduced by the FDGM and is illustrated in Figure 3.

At this stage, it is important to stress that the energetic picture illustrated in Figure 3 is not intended to represent directional transport or anisotropic surface motion. Instead, it captures the global reshaping of the free-energy landscape induced by the external field, which increases the energetic cost of large-amplitude height fluctuations independently of their in-plane orientation. This perspective is fully consistent with the continuum analysis in Section 6, where the field-induced stabilization enters through an isotropic non-conservative relaxation channel. Directional effects associated with biased diffusion and attachment kinetics act at smaller length scales, while the suppression of long-wavelength roughness arises from a global energetic damping mechanism.

The dashed line marks $k/k_{c}=1$, where the diffusive and field-induced damping contributions are equal ($\nu k^{4}=\Gamma_{AC}$). Although the two curves become visually indistinguishable only for $k/k_{c}\gg 1$, the crossover scale is defined physically by this equality.

### 7.2. Nucleation as Energy-Barrier Minimization

At the atomic scale, nucleation corresponds to overcoming an energy barrier associated with the creation of new interfaces. In classical nucleation theory, this barrier is determined solely by surface energy and supersaturation. Within the FDGM, the field contribution lowers the total free energy of cluster configurations favorably oriented with respect to the field-induced energetic landscape, thereby reducing the effective nucleation barrier.

This modification follows directly from minimization of $F_{tot}$: configurations in which the dipolar contribution reduces the total energy are statistically favored. Field-assisted nucleation is therefore not an additional kinetic pathway, but the natural consequence of an altered energetic minimum.

### 7.3. Diffusion as Biased Descent in a Modified Energy Landscape

Surface diffusion can be viewed as a stochastic exploration of the local energy landscape by adatoms [5,6]. In the absence of fields, this landscape is isotropic, and diffusion proceeds with equal probability along symmetry-equivalent directions. The introduction of $F_{field}$ breaks this isotropy by tilting the energy landscape in field-favored directions.

As shown in Section 4, the resulting anisotropic diffusion coefficients emerge because adatom hops that reduce the field-coupled energy experience lower activation barriers. Diffusion thus follows the steepest descent of the modified free energy, providing a direct link between microscopic field–matter interactions and mesoscopic mass transport.

### 7.4. Coalescence as Collective Energy Reduction

At the mesoscale, islands represent collective degrees of freedom whose interactions are governed by surface, strain and field energies. Directed coalescence arises when island configurations that minimize the combined energetic contributions are preferentially selected during growth, consistent with energetic descriptions of island coalescence and coarsening in thin films [5,6,18].

From the unified perspective, the field-induced biases in island motion and reshaping can be interpreted as reflecting gradients of $F_{tot}$ in the space of island configurations and attachment pathways, rather than as literal mechanical forces acting on islands in a uniform field. Texture selection, densification and aligned grain boundaries are therefore emergent consequences of collective energy minimization under an externally modified landscape, mediated by anisotropic attachment–detachment kinetics and biased mass transport during coalescence.

### 7.5. Morphological Stability as Global Energy Damping

At the continuum scale, deviations of the surface profile from an energetically optimal configuration increase both surface energy and field-coupled energy. The additional stabilizing term derived in Section 6 reflects the system’s tendency to suppress such deviations through an effective non-conservative energetic damping channel.

The field-induced damping of long-wavelength surface modes can thus be interpreted as a global energetic penalty for large-scale height fluctuations. This mechanism enforces convergence toward a morphology that minimizes $F_{tot}$ at the macroscopic level, complementing the local minimization processes governing nucleation, diffusion and coalescence, in direct analogy with energetic stabilization mechanisms in classical theories of surface relaxation and morphological stability [5,6,12].

### 7.6. Multiscale Coherence of the FDGM

Taken together, these arguments establish that the FDGM is not a collection of independent or phenomenological field-induced effects, but a coherent multiscale theory. The same energetic term that biases atomic nucleation also governs diffusion anisotropy, directs island coalescence and stabilizes continuum surface modes. Each growth stage can therefore be understood as a scale-specific expression of the same underlying variational principle.

This multiscale coherence distinguishes the FDGM from phenomenological models in which field effects are introduced separately at different stages of growth. By grounding all field-driven phenomena in a single free-energy functional, the FDGM provides conceptual clarity and a basis for predictive modelling.

## 8. Manufacturing Implications for Thin-Film-Based Devices

Although the Field-Driven Growth Model (FDGM) is formulated as a general theoretical framework, its implications are directly relevant to the manufacturing of advanced thin-film-based devices. By providing explicit energetic mechanisms and analytical stability criteria, the FDGM enables a shift from empirical optimization toward a more predictive and physically grounded control of thin-film growth processes [2,8,18].

### 8.1. Control of Nucleation Density Without Chemical Modification

In many manufacturing contexts, control of nucleation density is achieved by adjusting precursor chemistry, deposition rate or substrate temperature. These approaches often involve trade-offs between uniformity, throughput and compatibility with device integration. Within the FDGM, external fields provide an alternative control parameter: the nucleation barrier can, in appropriate regimes, be reduced directly through field–matter coupling, without modifying chemical composition or thermal budgets [1,2,18].

This mechanism is particularly relevant for atomic layer deposition and chemical vapour deposition, where uniform nucleation across large areas and high-aspect-ratio structures is critical. Field-assisted nucleation offers a route to achieve conformal coatings and reduced incubation times while preserving process compatibility with temperature-sensitive substrates [1,2].

### 8.2. Engineering Diffusion and Texture via Field-Controlled Anisotropy

Surface diffusion strongly influences grain size, texture and surface roughness in physical vapour deposition techniques such as sputtering and pulsed-laser deposition. Classical strategies to control diffusion rely on substrate heating or intrinsic material anisotropy, both of which are often difficult to tune independently [5,6,18].

The FDGM predicts that anisotropic diffusion can be externally controlled through the applied field, enabling directional mass transport without altering substrate temperature. This capability provides a theoretical basis for experimentally observed field-induced texture selection and grain alignment, and suggests that external fields may be used as an additional process knob to engineer microstructure in thin films for electronic, optical and sensing applications [8,18]. Recent experimental work demonstrates that externally applied electric fields can significantly influence growth kinetics, morphology and functional properties of thin films. For example, controlled electric fields during chemical bath deposition modify ZnO film structure and optical response, while field-assisted deposition of carbon nanoparticle films alters morphology and electrical performance. These observations are consistent with field-driven energetic effects acting across different material systems and deposition techniques [9,19].

### 8.3. Suppression of Large-Scale Roughness Through Field-Induced Stabilization

Surface roughness at length scales comparable to device feature sizes can significantly degrade device performance, particularly in multilayer stacks and interfacial layers. Classical surface-diffusion-driven relaxation suppresses roughness only slowly at long wavelengths, limiting achievable smoothness during continuous growth [5,6]. Similar energetic–morphological correlations have been widely discussed in energetic condensation and plasma-assisted deposition contexts, where increased energy flux is known to suppress large-scale roughening and promote densification [20].

While energetic condensation and plasma-assisted growth achieve roughness suppression through increased kinetic energy flux and localized energetic events, the stabilization mechanism predicted by the FDGM arises from an explicit field–matter interaction that introduces a persistent restoring contribution to the free-energy landscape. Although both approaches lead to enhanced morphological uniformity, their physical origins and control parameters are fundamentally distinct, with the FDGM explicitly addressing field–matter coupling rather than kinetic energy flux effects.

Within the FDGM, the existence of a field-controlled stability cutoff wavelength establishes a quantitative criterion for suppressing long-wavelength roughening modes. By operating in regimes where the field-induced stabilization dominates over curvature-driven relaxation, manufacturing processes can actively damp surface fluctuations that would otherwise persist. This mechanism provides a direct route towards thin films with improved uniformity without resorting to post-deposition planarization [5,6,12].

### 8.4. Qualitative Experimental Illustration

**Figure 4:**
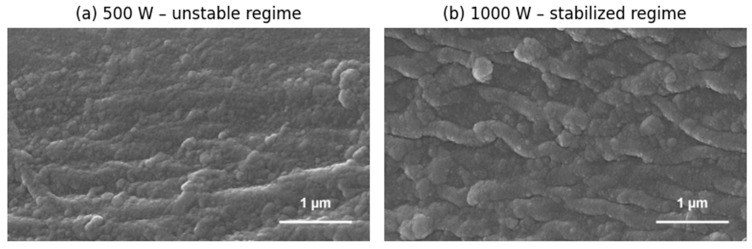
Representative SEM images of TiO_2_ thin films deposited by reactive DC magnetron sputtering under identical conditions, except for the applied power: (**a**) 500 W, corresponding to a growth regime with pronounced long-wavelength morphological modulations; (**b**) 1000 W, corresponding to a more compact morphology with reduced amplitude of large-scale surface undulations. The images are shown as a qualitative illustration of roughness suppression under more stabilizing energetic conditions and do not constitute a direct validation of field-induced isotropic or directional stabilization. For completeness, the films were deposited onto lignocellulosic fibre substrates at near-room temperature using an Ar/O_2_ reactive atmosphere under a total pressure of a few pascals, with the oxygen partial pressure adjusted to ensure stoichiometric TiO_2_ growth, as described in detail in ref. [21].

Although the Field-Driven Growth Model (FDGM) is formulated as a general theoretical framework, it is instructive to compare its qualitative predictions with representative morphological trends observed experimentally, without implying a direct one-to-one correspondence. While the applied power modifies the overall energetic conditions of growth rather than introducing a controlled external field, the resulting morphological trends provide a useful qualitative illustration of the stabilization mechanisms captured by the FDGM. Figure 4 shows SEM images of TiO_2_ thin films deposited by reactive DC magnetron sputtering under identical conditions, except for the applied power. The experimental procedure follows that described in detail in [21]. At 500 W, the surface exhibits pronounced long-wavelength morphological modulations characteristic of an energetically unstable growth regime. At 1000 W, the morphology becomes more compact, and the amplitude of large-scale surface undulations is reduced, consistent with enhanced damping of long-wavelength modes. We note that in-plane texture or elongation may persist in both cases due to deposition geometry and plasma asymmetries inherent to magnetron sputtering. Accordingly, Figure 4 is presented solely as a qualitative illustration of large-scale roughness suppression under more stabilizing energetic conditions, and not as evidence of isotropic or directional field effects.

### 8.5. Process Integration and Scalability

An important advantage of field-assisted growth strategies is their compatibility with existing deposition platforms. External electric (and, in specific material systems, magnetic) fields can often be implemented through substrate biasing, electrode design or magnetic field coils, without substantial modification of reactor geometry or chemistry [2,8,18].

The FDGM provides guidance on how such fields influence growth across length scales, enabling rational selection of field strength, orientation and temporal modulation to target specific morphological outcomes. Because the underlying energetic mechanisms are general, the framework applies across a wide range of materials systems and deposition techniques, supporting scalability from laboratory to industrial manufacturing environments [1,2,18].

Within this context, it is useful to delineate the typical process window over which the assumptions of the FDGM are expected to hold. The present framework applies to moderate external electric fields, such as those generated by substrate biasing, sheath fields, or electrostatic charging in plasma-assisted deposition, where linear polarization and weak field–matter coupling remain valid. In practical terms, this corresponds to field strengths commonly encountered in sputtering, PLD, CVD and ALD environments, where external fields influence surface energetics without inducing dielectric breakdown, field emission, or strongly nonlinear plasma effects. Outside this regime—such as at very high field intensities or under strongly non-equilibrium plasma conditions—additional nonlinear or electrodynamic contributions may become relevant, as discussed in Section 9.5.

### 8.6. Implications for Thin-Film-Based Device Performance

By linking field parameters to nucleation density, diffusion pathways and morphological stability, the FDGM establishes a physically motivated connection between process conditions and device-relevant film properties. Improved surface uniformity, controlled texture and reduced defect density translate into enhanced electrical performance, optical quality and mechanical reliability in thin-film-based devices [2,8,18].

In this sense, the FDGM does not merely rationalize observed field effects, but provides a physically motivated and predictive theoretical framework for integrating external fields as active control parameters in the manufacturing of next-generation thin-film-based technologies [1,2,18].

## 9. Discussion

The Field-Driven Growth Model proposed in this work provides a unified theoretical framework for understanding how external electric (and, in specific material systems, magnetic) fields influence thin-film growth across multiple length scales. In this section, we discuss the implications of the FDGM in the context of existing growth theories, clarify its domain of validity, and highlight its conceptual and practical significance.

### 9.1. Positioning the FDGM Within Classical Growth Theory

Classical thin-film growth models have been remarkably successful in describing a wide range of phenomena under intrinsic growth conditions. Burton–Cabrera–Frank step-flow theory accurately captures terrace and step dynamics during epitaxial growth, classical nucleation theory describes island formation as a balance between surface energy and supersaturation, and Mullins-type models explain curvature-driven surface relaxation through surface diffusion. However, a common and fundamental assumption underlying all these approaches is that the energetic landscape governing growth is unaffected by external fields under intrinsic growth conditions [3,4,5,6].

The FDGM departs from this assumption by explicitly incorporating field–matter interactions into the free-energy functional. This modification does not invalidate classical theories; rather, it extends them. In the absence of external fields, the FDGM reduces naturally to standard growth models. When fields are present, the same energetic framework predicts additional field-dependent energetic contributions that bias nucleation, diffusion, coalescence and morphological stability in a consistent manner. This continuity with established theory is an important strength of the model [3,5,6]. Importantly, the FDGM does not introduce field effects as independent phenomenological corrections at different stages of growth. Instead, all field-driven phenomena emerge consistently from the same free-energy functional, ensuring multiscale coherence and internal consistency. This distinguishes the FDGM from earlier approaches, where field effects were often treated in an informal or scale-specific manner.

### 9.2. Comparison with Edwards–Wilkinson and KPZ Frameworks

Continuum growth models such as the Edwards–Wilkinson (EW) and Kardar–Parisi–Zhang (KPZ) equations have played a central role in understanding kinetic roughening and scaling behavior of growing interfaces [6,10]. The EW equation describes linear diffusive relaxation with stochastic noise, while the KPZ equation introduces a nonlinear term associated with lateral growth, leading to non-Gaussian scaling and roughening.

Within these frameworks, anisotropy and instability arise from intrinsic surface properties, nonlinearities or noise, rather than from externally controllable parameters. Although extensions of EW and KPZ can include anisotropic coefficients, such anisotropy is typically static and material-specific. In contrast, the FDGM introduces an externally tunable stabilization mechanism that directly suppresses long-wavelength roughening modes through a field-controlled energetic term. This mechanism is fundamentally different from the nonlinear roughening captured by KPZ-type dynamics and cannot be reproduced by adjusting kinetic coefficients alone.

From a universality perspective, the FDGM suggests the existence of a field-driven growth regime in which external fields alter the effective scaling behavior of the interface. While a full renormalization-group analysis is beyond the scope of the present work, the analytical suppression of long-wavelength modes indicates that field-assisted growth may not be fully captured within traditional EW or KPZ universality classes [6,10]. Recent phase-field modelling studies likewise show that external electric fields can reshape the energetic landscape governing thin-film crystallization [17] and thin-film phase stability/domain evolution, yielding field-controlled morphological outcomes [22].

### 9.3. Relation to Slope-Dependent Diffusion Models

The Wolf–Villain model and related slope-dependent diffusion frameworks [11] capture important aspects of mound formation and kinetic roughening by introducing diffusion currents that depend on local surface slope. These models successfully describe intrinsic instabilities arising from surface geometry and step-edge barriers.

However, slope-dependent diffusion models treat anisotropy as an emergent property of the surface itself, not as an externally controllable parameter. In contrast, the anisotropic diffusion predicted by the FDGM arises directly from field–matter coupling and can be dynamically tuned through the magnitude, orientation or temporal modulation of the applied field. This distinction is critical for manufacturing applications, where external control parameters offer greater flexibility than intrinsic material constraints [11], and where energetic rather than purely geometric anisotropy is experimentally accessible.

### 9.4. Multiscale Coherence and Predictive Capability

A central contribution of the FDGM is its multiscale coherence. The same field-dependent energetic term governs atomic-scale nucleation, mesoscale diffusion and coalescence, and continuum-scale morphological stability. This coherence addresses a long-standing gap in the literature, where field-induced effects are often explained independently at different scales using unrelated mechanisms in the existing thin-film growth literature [10,11].

By grounding all field effects in a single free-energy functional, the FDGM provides a predictive theoretical basis, rather than purely post hoc interpretation. The analytical stability condition derived for continuum surface modes offers a concrete example: it links measurable process parameters to morphological outcomes in a way that is not accessible within classical growth models [5,6,10,11]. Because the FDGM is formulated at the level of a general energetic functional, its predictions are not tied to a specific experimental configuration or deposition technique. Instead, the model yields analytical stability criteria and scaling relations that can be directly mapped onto experimentally accessible process parameters. In this sense, the FDGM is intended as a predictive framework that guides experimental design and process optimization, rather than a model calibrated to reproduce a particular dataset.

### 9.5. Limitations and Scope of Applicability

Like any theoretical framework, the FDGM has limitations. The model assumes moderate field strengths, where linear polarization and dipolar coupling provide an adequate description of field–matter interactions, and where the dominant energetic contributions can be consistently expanded to leading order in the field amplitude. At very high field intensities, nonlinear effects, field-induced desorption or plasma-specific phenomena may become important and require additional terms [10,11].

Furthermore, the continuum analysis neglects explicit nonlinearities in the surface evolution equation, such as those associated with strong lateral growth or shadowing effects. While the present formulation captures the dominant stabilizing influence of external fields, future extensions may incorporate nonlinear terms to explore the interplay between field-induced damping and kinetic roughening in greater detail [6,10].

While the present formulation is most directly applicable to electric fields acting on polarizable surface species, extension to magnetic fields requires the presence of magnetic moments or susceptibilities and is therefore material-dependent.

In addition, the FDGM describes an averaged, statistical growth regime appropriate for continuum and mesoscale descriptions, in which external fields act primarily by modifying energetic barriers and introducing weak but persistent restoring contributions. While recent experimental and modelling studies confirm that such mechanisms operate under realistic deposition conditions [8,14,15,16], and that related field-induced energetic effects can influence post-growth structural stability in thin films [17], the model is not intended to capture growth phenomena dominated by highly localized high-energy events, electrical breakdown, or strongly nonlinear plasma effects. In such regimes, a more detailed kinetic, plasma-specific or electrodynamic description would be required to account for transient, non-equilibrium processes beyond the scope of the present energetic framework.

The FDGM is formulated as a general framework and does not explicitly account for material-specific electronic or magnetic properties. Incorporating such effects may further refine predictions for particular materials systems, but does not alter the core energetic principle underlying the model [10,11].

Several of the limitations of the present formulation can be systematically addressed in future extensions of the FDGM. Strong-field regimes may be incorporated by retaining higher-order polarization terms in the free-energy functional, while nonlinear surface evolution can be treated by augmenting the continuum dynamics with KPZ-type or shadowing terms. Material-specific electronic or magnetic effects can be introduced through appropriate constitutive relations for the polarization or magnetization response.

### 9.6. Broader Implications

Despite these limitations, the FDGM offers a new perspective on field-assisted thin-film growth. It explains why similar field-induced ordering phenomena are observed across disparate deposition techniques and materials systems [1,2,8,18], and it provides a conceptual foundation for treating external fields as active control parameters rather than secondary process conditions.

By unifying nucleation, diffusion, coalescence and stability within a single energetic framework, the FDGM bridges the gap between empirical observations and predictive manufacturing theory. This positioning suggests that field-assisted growth should be viewed not merely as a special case of classical thin-film deposition, but as a distinct regime of non-equilibrium growth with its own governing principles.

## 10. Conclusions

In this work, we have introduced the Field-Driven Growth Model (FDGM) as a unified theoretical framework for thin-film growth under external electric fields (and, in material-dependent cases, magnetic fields). By explicitly incorporating field–matter interactions into the free-energy functional governing growth, the FDGM provides a physically grounded and internally consistent description of how external fields influence thin-film morphology across multiple length scales.

Starting from a general energetic formulation, we showed that the inclusion of a field-dependent term modifies the fundamental processes of nucleation, surface diffusion and island coalescence in a coherent manner. Field-assisted nucleation emerges naturally from a field-dependent reduction in the nucleation barrier, anisotropic surface diffusion arises from direction-dependent modification of diffusion activation energies, and directed coalescence results from field-induced energetic biases at the mesoscale. At the continuum level, these effects culminate in a field-induced stabilization mechanism that suppresses long-wavelength roughening modes and defines a field-controlled morphological stability cutoff.

A central contribution of the FDGM is the demonstration that these apparently distinct phenomena are not independent mechanisms, but multiscale manifestations of a single underlying principle: the minimization of a field-modified free-energy landscape under non-equilibrium growth conditions. This multiscale coherence distinguishes the FDGM from phenomenological or scale-specific models and provides a transparent physical interpretation of field-assisted growth.

By deriving explicit analytical stability conditions and linking them to measurable process parameters, the FDGM offers predictive capability that is absent from classical growth theories and their extensions. In particular, the identification of a field-controlled cutoff wavelength provides a quantitative criterion for suppressing large-scale roughness during growth, with direct implications for the manufacturing of uniform thin films.

The framework developed here is broadly applicable to a wide range of deposition techniques, including sputtering, pulsed-laser deposition, chemical vapor deposition and atomic layer deposition. By treating external fields as active control parameters rather than secondary perturbations, the FDGM establishes a theoretical foundation for rational design of field-assisted manufacturing strategies in advanced thin-film-based devices.

Within this framework, the FDGM enables targeted control and improvement of key thin-film growth properties, including nucleation density and spatial uniformity, long-wavelength surface roughness, grain size distribution and texture, film densification, and defect reduction. By explicitly linking these properties to external field parameters through analytical stability criteria, the model provides predictive guidance that is not accessible within classical field-free growth theories.

Future work may extend the FDGM to include nonlinear surface evolution terms, strong-field regimes and explicit material-specific electronic or magnetic responses, and to explore scaling behavior through numerical simulations. Nevertheless, the present formulation already captures the essential physics underlying field-driven thin-film growth and provides a robust basis for both experimental interpretation and process optimization.

## Figures and Tables

**Table 1:** Key FDGM parameters.

Parameter	Meaning	Units/Scaling in the Model	Controls/Measurable via
E	Applied electric field	$[E]=V\;m^{-1}$; enters typically as *E*(drift) and/or $E^{2}$ (induced effects)	Set mainly by substrate bias and sheath; affects J(E) and $\Gamma_{AC}$
α	Effective polarizability of adatom/cluster	$[\alpha ]={\mathrm{Cm}}^{2}\;V^{-1};\;\mathrm{contributes}\;\Delta U∼-\frac{1}{2}\alpha E^{2}$	Inferred from material response; effective value may increase for clusters
p_eff_	Effective barrier-bias parameter	“Barrier-bias” coefficient; enters $\Delta G^{*}(E)$ and thus J(E)	Fitted from nucleation statistics vs. bias/field (nucleation rate, island density)
β	Diffusion–field coupling	coupling controlling field-driven/anisotropic diffusion, e.g., D(θ,E)	Extracted from tracer measurements and/or PSD anisotropy under bias
ν	Surface-diffusion smoothing coefficient	$[\nu ]\;=\;m^{4}\;s^{-1};\;\mathrm{sets}\;k^{4}$-type relaxation	Estimated from surface diffusion parameters and capillarity (e.g., $D_{s},\;\gamma ,\;\Omega ,\;T$); also fit from coarsening/PSD dynamics
Γ_AC_	Field-induced relaxation rate	$[\Gamma_{\mathrm{AC}}]=s^{-1};\;\mathrm{often}\;\propto E^{2}$ in linear response; defines $k_{c}=(\Gamma_{AC}/\nu {)}^{1/4}$	Extracted from PSD roll-off/crossover; controlled by bias and plasma conditions

## Data Availability

The original contributions presented in the study are included in the article, further inquiries can be directed to the corresponding author.

## Nomenclature

Symbol	Meaning	Units
h(r,t)	surface height field	m
r	in-plane position vector	m
t	time	s
E(t)	applied electric field	V m^−1^
P(r)	effective polarization density of near-surface species	—
α, α_e_	effective polarizability	C m^2^ V^−1^
ΔU	induced-dipole interaction energy	J
G(r)	cluster formation free energy (no field)	J
G_tot_(r)	cluster formation free energy (with field)	J
r	cluster radius	m
r_c_	critical radius	m
ΔG*	nucleation barrier (no field)	J
ΔG*_eff_	effective nucleation barrier (with field)	J
J(E)	nucleation rate (field-dependent)	—
p_eff_	effective barrier-bias parameter	J (V m^−1^)^−1^
λ	geometric factor (shape/partial wetting/orientation)	–
E_d_	diffusion barrier (no field)	J
E_d,eff_(θ)	field-modified diffusion barrier	J
β	diffusion–field coupling parameter	J (V m^−1^)^−1^
D_s_	surface diffusion constant	m^2^ s^−1^
D(θ,E)	anisotropic, field-dependent diffusivity	m^2^ s^−1^
γ	surface energy	J m^−2^
Ω	atomic volume	m^3^
k_B_	Boltzmann constant	J K^−1^
T	temperature	K
ν	surface-diffusion smoothing coefficient (prefactor of ∇^4^h)	m^4^ s^−1^
Γ_AC_	field-induced relaxation rate	s^−1^
η(r,t)	stochastic noise term	—
k	wavenumber	m^−1^
k_c_	crossover wavenumber, k_c_ = (Γ_AC_/ν)^1/4^	m^−1^
∇^2^, ∇^4^	Laplacian/biharmonic operator (in-plane)	m^−2^, m^−4^

## Appendix A. Why an Induced (Quadratic) Polarization Energy Can Be Represented as an Effective Directional Barrier Bias

In linear response, the polarization of a surface species can be written as P=α E, which yields the familiar induced-polarization energy

$$U_{pol}(E)=-\frac{1}{2}\alpha E^{2}.$$

Kinetics, however, depend on free-energy differences. The field-dependent contribution to the activation free energy is the difference between the transition-state (TS) and metastable initial (i) configurations:

$$\Delta G^{*}(E)-\Delta G^{*}(0)=[U^{TS}(E)-U^{i}(E)].$$

A convenient generic representation of the field-coupled energy of a configuration includes both an induced (quadratic) term and a possible effective dipolar (linear) term:

$$U(E)=-p\cdot E-\frac{1}{2}\alpha E^{2},$$

where p denotes an effective (possibly environment-induced) dipole moment and α an effective polarizability. Therefore, the barrier shift can be written as

$$\Delta G^{*}(E)=\Delta G^{*}(0)-\Delta p\cdot E-\frac{1}{2}\Delta \alpha \;E^{2},$$

with $\Delta p=p^{TS}-p^{i}$ and $\Delta \alpha =\alpha^{TS}-\alpha^{i}$. Differences in p and/or α naturally arise because the TS and the initial configuration can have different local bonding, charge redistribution, geometry, or orientation relative to the field.

For practical modeling over a restricted process window (i.e., a limited range of applied fields relevant to thin-film manufacturing), it is often useful to recast the field dependence into a compact effective directional bias of the form used in Equations (9) and (10):

$$\Delta G^{*}(E)\approx \Delta G^{*}(0)-\lambda \;p_{eff}\cdot E,$$

where λ is a geometric factor (shape/orientation/partial wetting) and $p_{eff}$ absorbs the microscopic pathway dependence. In particular, one may interpret

$$p_{eff}(E)=\Delta p+\frac{1}{2}\Delta \alpha \;E,$$

and, over a narrow field range around a representative operating point $E_{0}$, treat $p_{eff}$ as approximately constant (or equivalently, linearize the quadratic contribution about $E_{0}$). Importantly, this representation does not claim that an induced dipole in a strictly uniform field produces a fundamental linear coupling; rather, it is an effective barrier-bias parametrization of field-induced asymmetry between competing nucleation pathways.
